# Repeatable individual variation in migration timing in two anadromous salmonids and ecological consequences

**DOI:** 10.1002/ece3.6808

**Published:** 2020-09-24

**Authors:** Arne Johan Jensen, Bengt Finstad, Peder Fiske, Ola H. Diserud, Eva B. Thorstad

**Affiliations:** ^1^ Norwegian Institute for Nature Research (NINA) Trondheim Norway; ^2^ Department of Biology Norwegian University of Science and Technology (NTNU) Trondheim Norway

**Keywords:** anadromous, behavior, individual variation, migration, repeatability, *Salmo*, *Salvelinus*

## Abstract

Consistent individual differences in behavior have been demonstrated for many animals, but there are few studies of consequences of such repeated behavior in the wild. We tested consistency in migration timing to and from the sea among anadromous Arctic char (*Salvelinus alpinus*) and brown trout (*Salmo trutta*), using data from a study period of about 25 years, including more than 27,000 uniquely Carlin‐tagged individuals that migrated to sea for feeding in the spring and returned to the river in late summer for up to 13 successive years. Consistency was found between individuals across time in timing of the seaward migration. Individuals migrating early during their first migration tended to migrate early the following years, and late migrants tended to migrate late. The same pattern was found also at ascent to freshwater. Hence, this study demonstrated that individual fish in nature can differ in behavior related to migration timing and that these differences can be consistent during their lifetime. Early migrants increased their mass more than late migrants and had a higher specific growth rate. Early migrating Arctic char, but not brown trout, experienced a longer life after the first migration to sea than late migrants. In both species, maturity occurred earlier in individuals that migrated early. For brown trout, but not for Arctic char, fecundity was significantly correlated to the timing of smolt migration. Hence, the repeatable individual variation in migration timing seemed to have ecological and fitness consequences in terms of growth, longevity, timing of maturity, and lifetime fecundity.

## Graphical abstract

This 25‐year study tested consistency in migration timing to and from the sea among anadromous Arctic char and brown trout, including more than 27,000 uniquely tagged individuals that migrated to sea for feeding in the spring and returned to the river in late summer. Repeatable individual variation in migration was found between individuals across time in timing of the seaward migration. Individuals migrating early during their first migration continued to migrate early the following years, and late migrants continued to migrate late. The same pattern was found also at ascent to freshwater. The personality differences in migration timing seemed to have ecological and fitness consequences in terms of growth, timing of maturity, and lifetime fecundity.

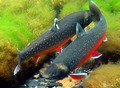

## 1 INTRODUCTION

Consistent individual differences in behavior have been demonstrated for a wide range of animals, such as insects (Stanley et al., 2017), lizards (Carter et al., 2012), birds (Schuett & Dall, 2009), mammals (Carter et al., 2012), and fishes (Coleman & Wilson, 1998; Conrad et al., 2011; Jolles et al., 2016; Mittelbach et al., 2014). This has been demonstrated for several types of behavior, including boldness, aggressiveness, activity, foraging, migration, mate choice, parental care, social network position, and leadership (Barbasch & Buston, 2018; Conrad et al., 2011; Mittelbach et al., 2014; Sih et al., 2012). Most studies of consistent individual differences in behavior have been conducted on animals in captivity under simplified and controlled conditions, while studies under seminatural and natural conditions are fewer (Dingemanse & Réale, 2005; Mittelbach et al., 2014; Réale et al., 2007; Sih et al., 2012).

Fishes have frequently been used as model organisms in behavior studies, and consistent maintenance of behavioral types has been documented in a variety of species. Most studies have been on shyness versus boldness, exploration, avoidance, activity, aggressiveness, and sociability (Conrad et al., 2011; Mittelbach et al., 2014). In recent years, several studies of fishes in the wild have demonstrated consistent differences in aspects of their migration behavior (Eldøy et al., 2019; Harrison et al., 2015; Tibblin et al., 2016; Villegas‐Ríos et al., 2018). Few studies have examined the ecological consequences of repeatability in behaviors in wild fish, especially under field conditions (Conrad et al., 2011; Mittelbach et al., 2014).

Migration is a strategy performed by many fishes and other animals to increase individual fitness (Chapman et al., 2012; Dingle & Drake, 2007). Individuals utilize the best‐suited habitat during different life stages to increase individual fitness by performing spawning and feeding migrations, or migrations may be a means of avoiding hostile conditions or seeking food resources. Seasonal migrations may evolve when the use of multiple habitats results in increased lifetime fitness (Gross et al., 1988). Migrations must be properly timed to optimize the balance between costs and benefits in terms of avoiding hostile conditions and utilizing resource availability to maximize fitness (Dingle & Drake, 2007; Jensen et al., 2019). In partially migratory fishes, bold individuals seemed more likely to migrate than shy individuals (Chapman et al., 2011; Hulthén et al., 2017). Similar results have been found also for birds (Mettke‐Hofmann et al., 2005; Nilsson et al., 2010).

Salmonids spawn in freshwater, but individuals of many salmonid species perform feeding migrations to the sea (Gross et al., 1988), including Arctic char (*Salvelinus alpinus*) and brown trout (*Salmo trutta*). In northern Arctic char and brown trout populations, individuals with an anadromous lifestyle move to the sea for the first time as smolts at a size of about 12–30 cm and an age of about 2–12 years (Berg & Berg, 1987a; Jensen et al., 2012). They utilize near‐coastal areas as feeding grounds and often return to freshwater later the same summer to overwinter in freshwater. Thereafter, most individuals continue to migrate to the sea each summer and return to freshwater after some weeks at sea for the rest of their lives (Jensen et al., 2015). Migration timing within the season varies among individuals, watersheds, and years (Berg & Berg, 1987a; Jensen et al., 2012).

**FIGURE 1:**
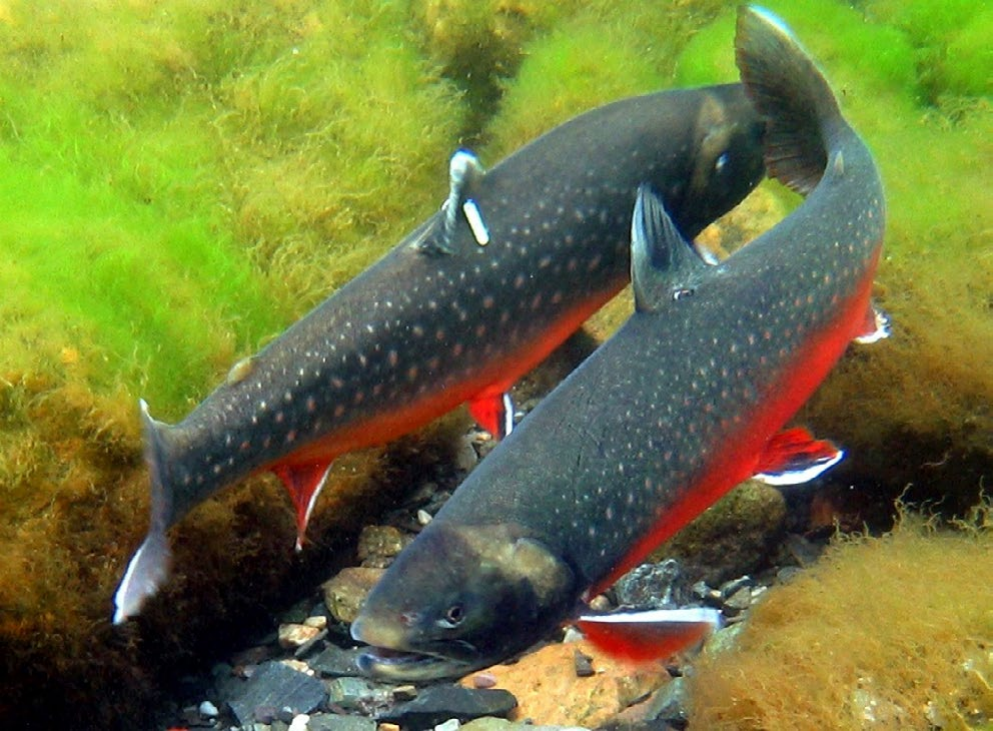
Two mature anadromous Arctic char from the Hals watershed, tagged with individual numbered Carlin tags attached just beneath the dorsal fin (Photograph: Svein Tore Nilsen)

In the present study, we tested consistency in migration timing to and from the sea among individuals of the River Halselva populations of anadromous Arctic char and brown trout. Furthermore, we studied ecological consequences of this repeatable individual variation in migration timing in terms of growth, survival, age at first maturity, and fecundity. The fish were individually tagged (Figure 1) and recorded when passing a trap in the river mouth. Following the first migration to sea, most individuals of both species returned to the river after some weeks in the sea, overwintered in freshwater, and continued to migrate between the river and the sea each summer for the rest of their lives. The study is unique in terms of covering the migration behavior of more than 27,000 individual fish and a study period of about 25 years. Some individuals were followed for up to 13 successive years, which is a longer‐lasting time period than in most other studies on repeatable individual migration. The aim of the study was to test consistency in individual variation in migration timing during migration to the sea and at return to the river, and ecological consequences of this repeatable individual variation in migration timing in terms of growth, survival, age at first maturity, and fecundity.

## 2 MATERIALS AND METHODS

### 2.1 Study area

**FIGURE 2:**
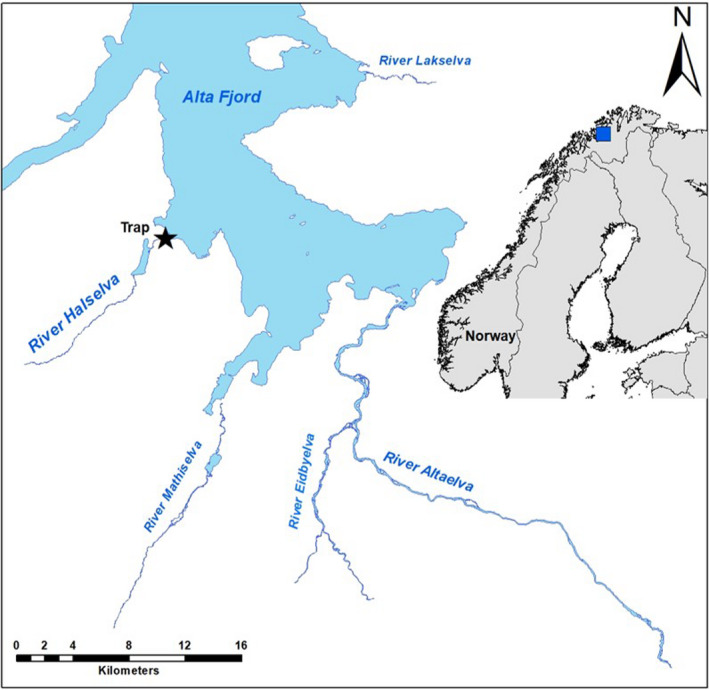
Map of the study area, with the location of the fish trap in the River Halselva for catching all ascending and descending fish

The Hals watershed in the Arctic region of Norway has a catchment area of 143 km^2^ and drains into the Alta Fjord at 70°2′N, 22°57′E (Figure 2). Approximately 20 km of the watershed is accessible to anadromous Arctic char, brown trout, and Atlantic salmon (*Salmo salar*), including a 1.2‐km^2^ lake located 2.1 km inland, 30 m above sea level (Lake Storvatn; Jensen et al., 2018b, Figure 2). The lake and river are ice‐covered from December to March or April, which is a period characterized by low water discharge. A pronounced increase in the discharge occurs during the snowmelt in May–June, followed by a decrease in July–August. Mean annual discharge is 4.3 m^3^/s. The River Halselva does not have a pronounced estuary and empties directly into the sea; hence, there are limited freshwater areas for fish to overwinter downstream of the fish traps (see below). Minimum water temperature at the outlet of the river is around 0°C during the ice‐covered period, after which the temperature increases to a maximum of approximately 13°C in early August. Minimum and maximum sea temperatures at 3 m depth are approximately 2.5°C in late March and 11°C during late July–early August (Jensen et al., 2018b).

### 2.2 Fish sampling

During 1987–2012, Arctic char and brown trout were sampled via permanent fish traps installed across the river 200 m upstream from the sea. All fish larger than 10 cm were trapped with a Wolf trap (Wolf, 1951) (apertures: 10 mm; inclination: 1:10) for descending fish and a fixed box trap for ascending fish (Jensen et al., 2018b). Arctic char and brown trout were predominant in the watershed, and Atlantic salmon and European eel (*Anguilla anguilla*) were present in low numbers. The traps were operated during the ice‐free period (April through October) and were emptied twice per day (at 0800 and 2000 hours). Body length (*L* in mm, measured as total length of the fish with the tail fin in its natural position, Ricker, 1979) and mass (*M*, in g) were recorded for all fish. Smolts (i.e., first‐time migrants, see definition by Allan & Ritter, 1977) of brown trout and Arctic char >18 cm were tagged with individually numbered Carlin tags (Carlin, 1955). These individuals were recorded each time they passed the fish traps during their annual migration to sea and back to the river for the rest of their lives. In general, smolts of Arctic char in this watershed migrate before brown trout, with median dates of descent of 25 June and 4 July, respectively, although some smolts of both species leave the river throughout most of the ice‐free period of the year (Jensen et al., 2012). Sex was determined by external inspection, and since this could be performed on mature individuals only, the sex distribution of immature individuals is unknown. Sex was determined for 10% of the Arctic char (132 males and 275 females) and 27% of the brown trout (453 males and 614 females). Arctic char and brown trout with a length of 18–28 cm that migrated to sea before 1 August in the period 1988–2012 (*n* = 11,900 Arctic char and 15,226 brown trout) were used to study individual variation in migration timing. Among these, 3,890 and 3,925 individuals, respectively, were recovered in the traps at least once.

For both species, there has been a substantial annual variation in migration timing in this watercourse (Jensen et al., 2012). To compensate for this, for each year and age‐group median dates for downward and upward migration as well as individual deviation from the median dates (i.e., standardized timing) were estimated.

The standardized mass‐specific growth rate (*Ω*, %/day) was used to eliminate the effect of differences in initial body sizes on growth rates (Finstad et al., 2011; Forseth et al., 2011; Jensen et al., 2018b; Sigourney et al., 2008) and was estimated according to Ostrovsky (1995) as follows:

$$\Omega =100\times \frac{M_{1}^{b}‐M_{0}^{b}}{\left(t_{1}‐t_{0}\right)\times b}$$

where *M_0_* is the body mass of the fish at descent from the river, *M_1_* the body mass of the same fish when returning to the river later the same year, *t_0_* the date when the fish descended, *t_1_* the date when the fish ascended again, *t*_1_ − *t_0_* the duration of the stay at sea, and *b* the allometric mass exponent for the relationship between specific growth rate and body mass (0.31 for brown trout, Elliott et al., 1995). The same value of *b* was used for Arctic char (Larsson et al., 2005).

Female fecundity was estimated for individuals as the number of eggs produced during their lifetime. Both species are iteroparous. After first maturity, they usually spawned each year until they died. First maturity usually occurred after 2–4 sea migrations in both species, with slightly lower mean sea age at maturity for Arctic char than for brown trout (Jensen et al., 2019). Fecundity (*b*) at a specific size was estimated according to Power et al. (2005) for Arctic char and Jonsson and Jonsson (1999) for brown trout. Power et al. (2005) estimated *b* in relation to fish length as follows:

$$b=0.625L^{2.224}$$

where *L* is fork length of the fish (in cm). To convert our length measurements to fork length, we used the factor 0.980 (unpublished data). Jonsson and Jonsson (1999) analyzed the relationship between *b* and mass (W) of first‐time and repeat spawners, respectively, of anadromous brown trout as follows:

lnb=0.800lnW+2.366

and

lnb=1.009lnW+0.695

As a mean for the period 1988–2012, 33.6% of Arctic char smolts survived the first migration to sea. After 2, 3, 4, 5, and 6 sea migrations, 10.8%, 6.3%, 3.3%, 1.9%, and 1.2% were still alive (Jensen et al., 2019). For brown trout, 28.1%, 14.4%, 10.2%, 6.0%, 2.9%, and 1.4% of the smolts were still alive after 1–6 sea migrations, respectively. Up to 13 sea migrations were observed for Arctic char and 11 for brown trout (Jensen et al., 2019).

### 2.3 Statistical analyses

All estimations and calculations were performed using the statistical software R (R Core Team, 2019). For the linear mixed modeling (LMM), we used the Ime4 package in R.

Repeatability (*R*) was estimated to examine whether differences among individuals in migration timing were consistent across years (Lessells & Boag, 1987; Nakagawa & Schielzeth, 2010; Sokal & Rohlf, 1981). The ANOVA‐based repeatability was calculated according to Nakagawa and Schielzeth (2010) as follows:

$$R=\frac{{\text{MS}}_{A}‐{\text{MS}}_{W}}{{\text{MS}}_{A}+\left(n_{0}‐1\right){\text{MS}}_{W}}$$

where MS*_A_* is the mean among‐individual sum of squares, and MS*_W_* is the within‐individual (residual) sum of squares. The correction term *n*_0_ was estimated as follows:

$$n_{0}=\frac{1}{k‐1}\left(N‐\frac{\sum_{i=1}^{k}n_{i}^{2}}{N}\right)$$

where *k* is the number of individuals, *N* is the total sample size, and *n_i_* is the sample size for the *i*th individual (Nakagawa & Schielzeth, 2010). Approximate confidence intervals for the ANOVA‐based repeatability estimates were calculated following Nakagawa and Schielzeth (2010). We obtained 10 and 8 separate repeatability estimates for Arctic char and brown trout, respectively, based on data for individuals that were captured in 2–13 different years. Each individual contributed with data to only one of the separate estimates. These ANOVA‐based repeatability estimates were compared with an overall repeatability estimated by a linear mixed model (LMM) (Dingemanse & Dochtermann, 2013), treating individual as a random factor. Information for all individuals with migration in the same direction registered for more than 1 year was used. Significance and confidence intervals for LMM‐based repeatability were estimated by 1,000 parametric bootstraps with the R‐package rptR (Stoffel et al., 2017).

## 3 RESULTS

### 3.1 Migration timing

**FIGURE 3:**
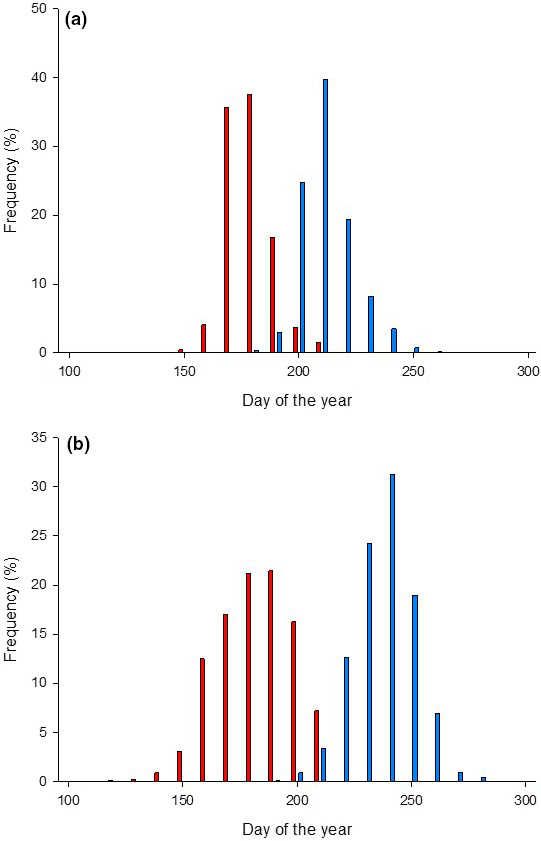
Seasonal variation in time over the period 1988–2012 when (a) Arctic char and (b) brown trout smolts migrated to sea from the River Halselva (red) and returned to freshwater (blue)

First‐time migrants of Arctic char descended earlier and returned to freshwater earlier than brown trout (Figure 3). Veteran migrants of both species migrated to sea and returned to freshwater earlier than first‐time migrants (Table 1). Both first‐time migrants and veteran migrants of brown trout stayed at sea for a longer time period than Arctic char (Table 1).

**Table 1:** Number of individuals (*N*) of Arctic char and brown trout descending to sea and returning to the River Halselva again (with median dates) during their first, second, third, and fourth sea migrations

	Period	Descent		Sea migration			Ascent	
		*N*	Median date	Days at sea	Growth increment	Ω	*N*	Median date
Arctic char	1st summer	11,900	25 June	33.7 ± 13.0	63.5 ± 47.8	7.19 ± 4.67	3,714	29 July
	2nd summer	1,475	16 June	35.3 ± 8.0	152.7 ± 61.8	10.33 ± 2.90	1,029	23 July
	3rd summer	715	13 June	34.2 ± 9.1	213.9 ± 80.7	10.23 ± 3.28	581	17 July
	4th summer	376	13 June	33.4 ± 7.5	249.7 ± 100.3	9.72 ± 2.74	313	15 July
Brown trout	1st summer	15,226	4 July	56.3 ± 14.3	156.2 ± 67.7	8.67 ± 2.78	2,796	26 August
	2nd summer	1,955	7 June	63.7 ± 22.4	317.2 ± 96.9	8.86 ± 2.01	1,281	8 August
	3rd summer	939	6 June	58.0 ± 19.9	457.8 ± 150.4	9.00 ± 2.56	1,041	29 July
	4th summer	708	3 June	56.2 ± 14.5	549.4 ± 187.0	8.83 ± 4.11	213	26 July

Note

The mean duration (±*SD*) of the sea migration (days at sea), growth increment (±*SD*) during the sea migration (g), and standardized mass‐specific growth rate (Ω, %/day) (±*SD*) are also given. Mean data for the period 1988–2012.

### 3.2 Sex and sexual maturation

First maturity usually took place 2–4 years after the first migration to sea, with a mean of 3.11 ± 0.11 (±95% CI) and 3.41 ± 0.05 years for Arctic char and brown trout, respectively. There was no significant difference between males and females in age at maturity in terms of number of years after the first sea migration (ANOVA tests, *F*_1_,_399_ = 1.693, *p* > .05 and *F*_1,1957_ = 0,944, *p* > .05, for Arctic char and brown trout, respectively). Males of Arctic char and brown trout migrated to the sea slightly earlier (2.8 and 0.6 days, respectively) than females (LMM tests with sex as fixed and individuals as random factor, *p* < .05 for Arctic char and *p* > .05 for brown trout). At ascent, no significant difference between the sexes in migration timing was found (*p* > .05). Sex was not taken into account in further analyses, since the differences in migration timing between males and females were small, and sex is known for only 10% of the Arctic char and 27% of the brown trout.

### 3.3 Repeatable individual variation in migration timing

Results showed repeatable individual variation in standardized migration timing, both at descent to the sea and return to the river (Tables 2, 3). Individuals migrating early to sea as smolts continued to migrate early also the following years. Overall repeatability for Arctic char estimated by LMM was 0.37 and 0.23 for descending and ascending individuals, respectively, which is comparable in magnitude to those obtained from one‐way ANOVAs (Table 2). For descending Arctic char, the one‐way ANOVA repeatability for *a* = 2, that is, for fish that are registered down the trap only as smolts and the year after, is lower than the overall repeatability. Repeatability for descending fish that have from 3 to 7 unidirectional migrations in total (i.e., groups with *a* from 3 to 7) increases and continues to have high values between 0.4 and 0.5, before decreasing for groups with *a* > 7. Repeatability for groups observed with migration in a given direction over more than 7 years will necessarily become uncertain, not only because there are few fish to estimate repeatability from, but also because the median date estimate required for the standardization of migration timing becomes more uncertain. For ascending Arctic char, the trend in one‐way ANOVA repeatabilities is the opposite, it starts at about the same value as for descending char but then decreases gradually up to *a* = 7.

**Table 2:** Consistency in standardized timing of descent to the sea and return to the river of Arctic char in the River Halselva across years

*a* (groups)	Descent to the sea					Return to the river				
	*n_0_*	*R*	*CI*	*N*	*p*	*n_0_*	*R*	*CI*	*N*	*p*
2	2.00	0.27	0.21–0.33	841	<.001	2.00	0.23	0.14–0.32	444	<.001
3	2.85	0.36	0.29–0.43	350	<.001	2.78	0.21	0.13–0.29	276	<.001
4	3.83	0.45	0.37–0.53	163	<.001	3.80	0.19	0.10–0.28	136	<.001
5	4.81	0.43	0.33–0.53	98	<.001	4.66	0.16	0.05–0.26	71	<.001
6	5.68	0.48	0.33–0.62	41	<.001	5.56	0.14	0.02–0.26	44	<.01
7	6.67	0.43	0.27–0.59	34	<.001	6.65	0.09	−0.03 to 0.21	32	<.05
8	7.86	0.15	‐	21	<.01	7.77	0.28	‐	13	<.001
9	8.57	0.29	‐	14	<.001	8.69	0.20	‐	13	<.001
10	10.00	0.04	‐	5	n.s.	9.74	0.22	‐	4	<.05
11	10.18	−0.02	‐	5	n.s.	10.38	0.20	‐	5	<.05
Overall		0.37	0.34–0.40	1,560	<.001		0.23	0.19–0.27	1,076	<.001

Note

*a* is the number of groups, *n_0_* is the number of measurements per individual, *R* is the repeatability estimate, *N* is number of individuals, *p* is the statistical significance, and *CI* is the 95% confidence interval for the repeatability. Overall repeatability was estimated from analysis of all data using LMM, according to Dingemanse and Dochtermann (2013).

**Table 3:** Consistency in standardized timing of descent to the sea and return to the river of brown trout in the River Halselva across years

*a* (groups)	Descent to the sea					Return to the river				
	*n_0_*	*R*	CI	*N*	*p*	*n_0_*	*R*	CI	*N*	*p*
2	2.00	0.12	0.06–0.17	1,137	<.001	2.00	0.30	0.22–0.38	514	<.001
3	2.77	0.08	0.02–0.14	482	<.01	2.07	0.27	0.19–0.34	593	<.001
4	3.06	0.10	0.04–0.16	394	<.001	2.19	0.18	0.10–0.26	448	<.001
5	3.36	0.14	0.08–0.20	341	<.001	3.19	0.26	0.17–0.34	206	<.001
6	3.99	0.19	0.10–0.27	147	<.001	3.53	0.11	0.01–0.22	104	<.01
7	4.67	0.20	0.09–0.31	69	<.001	4.19	0.26	0.10–0.42	44	<.001
8	5.04	0.09	–	29	n.s.	4.96	0.16	–	15	<.05
9	6.81	0.08	–	8	n.s.	NA	NA	–	2	NA
Overall		0.11	0.09–0.14	2,611	<.001		0.20	0.16–0.23	2057	<.001

Note

*a* is the number of groups, *n_0_* is the number of measurements per individual, *R* is the repeatability measurement, *N* is number of individuals, *p* is the statistical significance, and *CI* is the 95% confidence interval for the repeatability. Overall repeatability was estimated from analysis of all data using LMM, according to Dingemanse and Dochtermann (2013).

Overall repeatability for brown trout estimated by LMM was 0.11 and 0.20 for descending and ascending individuals, respectively (Table 3). For descending trout, the one‐way ANOVA repeatabilities have no apparent trend and are fairly low for all groups, although significant for *a* ≤ 7. The repeatabilities are higher for ascending trout than for descending, and for most groups slightly above those found for ascending char.

Several brown trout, and also a few Arctic char, overwintered one to four times in other watersheds before most of them returned to the River Halselva upon maturation (Jensen et al., 2015). Individuals with missing data for some years are included in the estimates presented in Tables 2 and 3. When these are excluded, the sample sizes decreased, but the overall repeatability estimates were almost the same and significant (Table S1).

In Arctic char, a highly significant relationship was found between individuals across time regarding the time they migrated to sea and returned to the river again (Table 4). Individuals migrating early (or late) to sea also returned to freshwater earlier (or later) than other individuals in the population. This was observed both the first year they migrated to sea and the following nine years (mean repeatability 0.54), although not significant the 8th summer at sea (Table 4). Similar behavior was observed also for brown trout the first three years (mean repeatability 0.55), but not later (Table 4).

**Table 4:** Repeatability (*R*) with 95% confidence interval (*CI*) between standardized timing of descent and ascent within the same summer for Arctic char and brown trout

Sea age		Arctic char				Brown trout				
	*R*	*CI*	*N*	*F*	*p*	*R*	*CI*	*N*	*F*	*p*
1	0.74	0.73–0.76	3,709	6.770	<.001	0.82	0.81–0.83	2,854	10.273	<.001
2	0.71	0.68–0.74	923	5.905	<.001	0.42	0.37–0.47	987	2.458	<.001
3	0.68	0.64–0.73	546	5.273	<.001	0.41	0.34–0.48	592	2.399	<.001
4	0.36	0.27–0.46	303	2.147	<.001	−0.04	–	370	0.930	n.s.
5	0.32	0.18–0.45	167	1.931	<.01	−0.02	–	226	0.963	n.s.
6	0.46	0.31–0.61	108	2.717	<.001	0.19	–	102	1.472	n.s.
7	0.32	0.09–0.55	64	1.944	<.05	−0.02	–	43	0.958	n.s.
8	0.07	–	36	1.142	n.s.	0.51	–	16	3.061	n.s.
9	0.93	–	22	25.911	<.001	0.43	–	6	2.500	n.s.
10	0.78	–	12	8.288	<.01	–	–	3		n.s.
11	−0.09	–	8	0.832	n.s.					

Note

The first 11 and 9 years after smoltification (sea age) were tested for Arctic char and brown trout, respectively. *N* is number of individuals, *F* is the *F*‐value, and *p* is the *p*‐values of one‐way ANOVA tests.

### 3.4 Ecological consequences of repeatable individual variation in migration timing

Individuals of both species migrating early to sea increased their mass during the sea migration to a greater extent than those migrating later (Table 5). Standardized mass‐specific growth rate was, however, best correlated with the date when they ascended to freshwater (Table 6). Individuals migrating early to freshwater had gained higher standardized mass‐specific growth rate than those returning later (Table 6).

**Table 5:** Pearson's correlation between timing of descent and ascent, respectively, and growth increment (in mass, g) during the 1st to 7th summer at sea for Arctic char and brown trout.

Period	Arctic char		Brown trout	
	Descent	Ascent	Descent	Ascent
1st summer	−0.372***	−0.058***	−0.515***	−0.054**
2nd summer	−0.460***	−0.262***	−0.163***	0.005
3rd summer	−0.566***	−0.222***	−0.095*	−0.003
4th summer	−0.547***	−0.130*	−0.168**	−0.053
5th summer	−0.329***	−0.211**	−0.188**	−0.027
6th summer	−0.382***	−0.184	−0.086	−0.007
7th summer	−0.164	−0.075	−0.114	0.272

* *p* < .05;

** *p* < .01;

*** *p* < .001.

**Table 6:** Pearson's correlation between timing of descent and ascent, respectively, and standardized mass‐specific growth rate (*Ω*, %/day) during the 1st to 7th summer at sea for Arctic char and brown trout

Period	Arctic char		Brown trout	
	Descent	Ascent	Descent	Ascent
1st summer	−0.255***	−0.205***	−0.070***	−0.035***
2nd summer	−0.030	−0.448***	0.150***	−0.414***
3rd summer	0.075	−0.167***	0.158***	−0.418***
4th summer	0.037	−0.290***	0.091	−0.316***
5th summer	−0.006	−0.256***	0.206**	−0.404***
6th summer	−0.072	−0.164	0.229*	−0.386***
7th summer	0.035	−0.067	0.208	−0.454**

* *p* < .05;

** *p* < .01;

*** *p* < .001.

A significant correlation was found between the time when Arctic char migrated to sea for the first time and survival (*r *= −0.108, *n* = 3,890, *p* < .001), suggesting that early migrating Arctic char experienced a longer life than their later migrating conspecifics. For brown trout, the opposite was found (*r* = 0.033, *n* = 3,924, *p* < .05).

Sea age at maturity was influenced by migration time in both species, although differently. In brown trout, maturity occurred at an older sea age in individuals that migrated late to the sea than in early migrating individuals (LMM with individuals as a random factor, *p* < .001), while timing of ascent was less important (*p* > .05). In Arctic char, however, timing of ascent was the most important, as individuals migrating early to freshwater matured significantly earlier than later migrating individuals (LMM with individuals as a random factor, *p* < .001). The relationship between timing of descent and age at maturity was also positive for Arctic char, but not significant (*p* > .05). However, standardized timing of migration explained only a small part of the variation in sea age at maturity. Individuals migrating one month (30 days) later are expected to become mature less than 0.16 and 0.40 years older in brown trout and Arctic char, respectively.

For brown trout, but not for Arctic char, a significant correlation between timing of smolt migration and total lifetime female fecundity was found (*r *= −0.109, *n* = 609, *p* < .01). The highest fecundity was found among early migrating smolts.

## 4 DISCUSSION

This study demonstrated that fish in nature can show individual variation in migration timing and that this can be consistent during their lifetime. Individuals migrating early during their first seaward migration tended to migrate early the following years, and late migrants tended to migrate late. The same pattern was found also when they returned to freshwater. Furthermore, individuals migrating early to sea also tended to return to the river earlier than their conspecifics.

The present results are in line with several other studies on individual repeatability of migration timing in animals, mainly birds (Bêty et al., 2004; Fraser et al., 2019; Lourenço et al., 2011) and fishes (Eldøy et al., 2019; Harrison et al., 2015; Tibblin et al., 2016). A common pattern seems to be that in partially migrating populations, bold individuals tend to migrate, while shy individuals tend to remain resident (Chapman et al., 2011; Mettke‐Hofmann et al., 2005; Nilsson et al., 2010). Furthermore, among migrating individuals, the bold ones tend to migrate first (Dingemanse & Réale, 2005; Tibblin et al., 2016), and there is evidence for a relationship between boldness and growth rate (Stamps, 2007; Ward et al., 2004). In our study, no information is available about boldness and shyness of individuals, but among first‐time migrants, the largest individuals tended to migrate first (Jensen et al., 2012), similar to bold individuals in the studies referred to above (Dingemanse & Réale, 2005; Tibblin et al., 2016).

The overall repeatability estimates in the present study (0.37 and 0.23 for descending and ascending Arctic char, and 0.11 and 0.20 for descending and ascending brown trout, respectively) are in the lower range of estimates summarized in a meta‐analysis of repeatability of behavior by Bell et al. (2009). The average repeatability across all estimates in the meta‐analysis, which included 759 estimates from 114 studies, was 0.37 (Bell et al., 2009). They found that the most repeatable classes of behavior were mating, habitat selection, and aggression, while the least repeatable behaviors were activity, mate preference, and migration. They also found that repeatability estimates were higher in the field compared to the laboratory and that repeatability was higher when the interval between observations was short (Bell et al., 2009). Although the present study was a field study, the interval between observations was as long as one year; hence, the lower than average repeatability in our study was as expected.

Tibblin et al. (2016) performed a 6‐year mark–recapture study, including 2048 individuals, of breeding migration in anadromous pike (*Esox lucius*). They found significant differences in standardized arrival time to the breeding site among individuals, and the individual differences were consistent across years. The overall repeatability (0.33 for females and 0.22 for males) was comparable in magnitude to that obtained for Arctic char and higher than for brown trout in the present study.

The overall repeatability of brown trout in the present study was higher for ascending than for descending fish, while the opposite was found for Arctic char. Higher repeatability for ascending than for descending brown trout was also found by Eldøy et al. (2019), who by telemetry studies over 3 years estimated a significant repeatability at return to the river, but not at migration to the sea.

The individual differences in migration timing in the present study seemed to have ecological and fitness consequences in terms of growth, longevity, timing of maturity, and lifetime fecundity. In general, the early migrants were the most successful in terms of growth and survival. Early migrants increased their mass more than late migrants and had a higher specific growth rate. Early migrating Arctic char, but not brown trout, experienced a significantly longer life after the first migration to sea than late migrants. For brown trout, but not for Arctic char, lifetime fecundity was significantly correlated with the timing of smolt migration, with early migrants producing a larger number of eggs during their lifetime. Research on fish has earlier shown that consistent individual differences within a single population can lead to contrasted reproductive success or survival (Cote et al., 2010; Smith & Blumstein, 2008).

Arctic char and sea trout typically migrate downriver to the sea in the spring, but the timing differs among regions and rivers, and even among years for each river (Jensen et al., 2012; Klemetsen et al., 2003). The timing of the seaward migration impacts post‐smolt survival in the marine environment, and it is believed that the fish use in‐river environmental cues that may predict favorable conditions in the sea to initiate downstream migration (Thorstad et al., 2012). There are many studies showing how in‐river factors stimulate the seaward migration in different populations of anadromous salmonids, reflecting different adaptations to ensure optimal conditions and high survival at sea entry (Hvidsten et al., 1995, 1998; Kallio‐Nyberg et al., 2006; McCormick et al., 1998). In the River Halselva, water flow had the most important impact on the timing of the brown trout smolt run, whereas photoperiod had the most important impact on the timing in Arctic char (Jensen et al., 2012). However, the duration of the migration period within a river in a given year may span over several weeks or months (Byrne et al., 2004; Jonsson & Jonsson, 2009; Pemberton, 1976). In the River Halselva, where this study was carried out, the time period from 25% to 75% of the smolts had left the river was on average 13 days in Arctic char and 28 days in brown trout (Jensen et al., 2012). The individual variation in timing of sea migration within a river in a given year, and what the causes and consequences are, has received little attention and is not well studied. In the present study, we show that individual differences in timing within a year may be linked to individual differences in fitness and hence are of great importance.

It is not surprising that early migration to sea was beneficial, because this is a period of the year that has been shown to have a rich prey fauna and is important for marine feeding and growth (Berg & Berg, 1987b; Knutsen et al., 2001, 2004; Olsen et al., 2006). However, it is also a period with high mortality risk due to predation and osmoregulatory problems (Klemetsen et al., 2003; Parker, 1971; Ward & Hvidsten, 2011), and the mortality is highly size‐selective, favoring large and fast‐growing individuals (Jensen et al., 2018a; Thorstad et al., 2016). Thus, because of the high mortality risk at this stage (Jensen et al., 2019), a trade‐off between energy gain and survival probably exists that maintains variation in migration timing. The largest smolts are the first ones to migrate to the sea, and the size of the smolts migrating to the sea decreases throughout the season, both in this river and in other rivers (Bohlin et al., 1993; Ewing et al., 1984; Jensen et al., 2012; Jutila & Jokikokko, 2008). One explanation may be that small individuals migrate to the sea in late summer because these individuals were too small to smoltify in the spring but reached a length suitable for smoltification later in the summer and hence migrated to the sea at a later date. Small individuals have poorer osmoregulation than larger individuals when reaching saltwater, particularly in cold water in the early spring (Klemetsen et al., 2003; Sigholt & Finstad, 1990). Further, the poorer swimming capacity of small individuals may imply a larger predation risk and lower sea survival compared to larger individuals. Hence, the large individuals may be able to migrate early and benefit from the growth opportunities at sea at a lower risk and cost than the small individuals, and large individuals may be favored both because they migrate early and because they are large. Veteran migrants, which have undertaken previous marine migrations, are larger than smolts. During springtime, larger and older veteran sea trout often descend to sea even earlier than the smolts (Berg & Jonsson, 1989; Jonsson & Jonsson, 2002, 2009; Pemberton, 1976), which was also shown in the present study.

It is not known to which extent the variation in migration timing discovered in the present study has a genetic versus an environmental basis. Arctic char and brown trout are variable and flexible species in terms of migration patterns and other life history traits, and both genes and environmental impacts may play a role. For instance, the decision to migrate to sea or remain in freshwater is partly genetically determined but is also a result of environmental conditions in freshwater and early life (Ferguson et al., 2019; Kendall et al., 2015). Around 50% of the variation in brown trout migration versus residency, among individuals within a population, is due to genetic variance (Ferguson et al., 2019). Differences between migrating and nonmigrating individuals of brown trout and Arctic char are often due to differences in energetic state, metabolic rate, growth rate, and lipid storage (Acolas et al., 2012; Ferguson et al., 2019; Strand & Heggberget, 1994). These factors may also influence the migration timing in these species. High metabolic requirements and growth rate during the parr stage may favor early smolt migration (Rikardsen & Elliott, 2000).

A striking result from the present study is how decisions early in life, or life history patterns from early on, persist during their lifetime, and may largely affect the lifetime success of individuals. When studying behavioral patterns in a natural population, the observed patterns and variation reflect the behavior of both the winners and losers in terms of fitness. The migrations between freshwater and the sea are life history events with a large natural mortality, particularly the first seaward migration as smolts, but also during later stages (Jensen et al., 2019; Kristensen et al., 2019). The results in the present study may support the silver spoon hypothesis, which proposes that individuals that develop under favorable conditions will gain fitness benefits throughout their lifetime (Grafen, 1988). Individuals that experience environmental conditions as juveniles in freshwater and/or with genes that contribute to a large smolt size and early smolt migration may benefit more from the growth season in the sea, enabling them to continue with early and longer migrations during following years, resulting in further growth benefits compared to the inferior individuals. This was contrasted by the results of Marco‐Rius et al. (2012), who found that freshwater growth was a poor predictor of final body size among sea trout populations in Spain. However, they sampled returning fish; hence, they only studied survivors, their study was a snapshot in time (one sampling season), and they studied fish that had remained at least one winter at sea, which may not be comparable to the fish spending only a few weeks or months at sea each year as they did in our study. Also, differences between productivity and growth potential in freshwater versus the sea and predation pressure may result in differences among sites and over time. Kallio‐Nyberg et al. (2006) found that the impact of sea trout smolt size on survival was condition‐dependent, because they found no correlation between smolt length and survival in good herring recruitment years, but in poor years, survival increased rapidly with increasing smolt size.

In brown trout, maturity occurred earlier in individuals that migrated early, and this may be connected to the growth/growth rate differences detected between early and late migrating individuals. Within brown trout populations, there is typically a negative correlation between early growth rate and age at maturity; the faster they grow, the younger they mature (Alm, 1959; Jonsson & Jonsson, 2011). In general, maturation should be initiated at the age when the expected fitness no longer increases by postponing maturity one more year (Jonsson & Jonsson, 2011). However, although statistically significant, the migration timing described only a small part of the variation in age at maturity. Age at maturity is phenotypically plastic and influenced by growth rate, size of energetic deposits, and the body size; hence, there may be individual, gender, population, and species differences in maturation responses to the same environmental stimuli, growth rates, body sizes, and stored energy reserves, depending on the fitness consequences of the variation (Jonsson & Jonsson, 2011).

The results in this study have consequences for the management of brown trout and Arctic char. In many areas in Norway, recreational fishing for brown trout in the sea in spring is popular, this fishery is not regulated, and there is no catch statistics available. If the early migrants are the most successful individuals, and these are to a larger extent than late migrants exploited, this may have consequences on the population level. Likewise, in the autumn, the duration of the fishing season for brown trout in the rivers varies, but often last till late August or mid‐September. Again, if the early migrants face a larger exploitation than those entering the rivers late, this may have consequences if these are among the most successful individuals.

The impact of the river and sea environments on the lifetime success of anadromous individuals is inevitably interlinked, where the conditions in one life stage and habitat impact later life stages. In this study of two partially migrating populations, where we only studied the sea‐migrating individuals, we could not compare the benefits of the observed migration strategies with those of freshwater‐resident individuals. Further, due to the tagging method and expected size‐selective tagging effects, the smaller individuals could not be included in the study. In future studies, it would be interesting to be able to include data on the freshwater‐resident part of the population and also to study the effects of the first years in the river, from the egg to smolt stage, on the life history decisions and success later in life. For an increased understanding of the population dynamics in such populations, and to be able to develop more efficient management and protection measures where needed, there is also need to understand the significance of the genetic versus environmental basis for the different life history decisions and the success at different life stages.

## CONFLICT OF INTEREST

None declared.

## AUTHOR CONTRIBUTION

**Arne Johan Jensen:** Conceptualization (equal); Data curation (lead); Formal analysis (lead); Investigation (equal); Methodology (equal); Project administration (lead); Resources (equal); Supervision (equal); Validation (equal); Writing‐original draft (lead); Writing‐review & editing (lead). **Bengt Finstad:** Conceptualization (supporting); Data curation (equal); Formal analysis (supporting); Methodology (equal); Project administration (supporting); Resources (supporting); Software (supporting); Validation (equal); Writing‐original draft (equal); Writing‐review & editing (equal). **Peder Fiske:** Conceptualization (supporting); Data curation (supporting); Formal analysis (equal); Funding acquisition (supporting); Investigation (supporting); Resources (supporting); Validation (equal); Writing‐original draft (equal); Writing‐review & editing (equal). **Ola Håvard Diserud:** Conceptualization (supporting); Formal analysis (equal); Investigation (supporting); Methodology (equal); Supervision (equal); Validation (equal); Writing‐original draft (equal); Writing‐review & editing (equal). **Eva Thorstad:** Conceptualization (equal); Data curation (supporting); Formal analysis (supporting); Investigation (supporting); Methodology (supporting); Validation (supporting); Writing‐original draft (equal); Writing‐review & editing (equal).

## Supporting information

Table S1

## Data Availability

The data are uploaded to Dryad (https://doi.org/10.5061/dryad.9s4mw6mdg).
