# Supplementary material for: Repeatable individual variation in migration timing in two anadromous salmonids and ecological consequences
Source: Ecol Evol. 2020 Sep 24;10(20):11727–38. doi: 10.1002/ece3.6808 (PMC7593174; doi:10.1002/ece3.6808)
Supplement: Supplementary file 1 — Table S1 [file ECE3-10-11727-s001.docx]

**Supplementary material**

**TABLE S1.** Consistency in standardized timing of migration to the sea and return to the river for a) Arctic char, and b) brown trout of the Halselva population across years. Only individuals that passed the trap each year until they disappeared are included. K is number of measurements per individual, R is the repeatability measurement, N is number of individuals, and P the statistical significance. Overall repeatability was estimated from analyses of all data using linear mixed modelling (LMM) and treating individual as a random factor (Dingemanse & Dochtermann 2013).

1. **Arctic char**

|  | Migration to sea | | |  | Return to the river | | |
| --- | --- | --- | --- | --- | --- | --- | --- |
| k (years) | *R* | N | P |  | *R* | N | P |
| 2 | 0.272 | 841 | <0.001 |  | 0.231 | 444 | <0.001 |
| 3 | 0.363 | 302 | <0.001 |  | 0.216 | 233 | <0.001 |
| 4 | 0.45 | 144 | <0.001 |  | 0.172 | 123 | <0.001 |
| 5 | 0.439 | 85 | <0.001 |  | 0.159 | 56 | <0.001 |
| 6 | 0.538 | 36 | <0.001 |  | 0.163 | 36 | <0.001 |
| 7 | 0.398 | 25 | <0.001 |  | 0.073 | 28 | <0.05 |
| 8 | 0.119 | 18 | <0.05 |  | 0.115 | 12 | <0.05 |
| 9 | 0.301 | 9 | <0.001 |  | 0.536 | 8 | <0.001 |
| 10 | 0.041 | 5 | n.s. |  | 0.295 | 3 | <0.05 |
| 11 | NA | NA | NA |  | NA | NA | NA |
| Overall | 0.373 | 1470 | <0.001 |  | 0.23 | 947 | <0.001 |

1. **Brown trout**

|  | Migration to sea | | |  | Return to the river | | |
| --- | --- | --- | --- | --- | --- | --- | --- |
| k (years) | *R* | N | P |  | *R* | N | P |
| 2 | 0.117 | 1137 | <0.001 |  | 0.296 | 514 | <0.001 |
| 3 | 0.069 | 400 | <0.01 |  | 0.213 | 278 | <0.001 |
| 4 | 0.092 | 169 | <0.01 |  | 0.058 | 112 | n.s. |
| 5 | 0.145 | 103 | <0.001 |  | 0.286 | 62 | <0.001 |
| 6 | 0.124 | 34 | <0.01 |  | -0.03 | 15 | n.s. |
| 7 | 0.084 | 9 | n.s. |  | 0.159 | 9 | <0.05 |
| 8 | 0.355 | 5 | <0.01 |  | NA | 2 | NA |
| 9 | NA | 2 | NA |  | NA | 2 | NA |
| Overall | 0.121 | 1955 | <0.001 |  | 0.192 | 994 | <0.001 |

Dingemanse N.J. & Dochtermann N.A. (2013). Quantifying individual variation in behaviour: mixed-effect modelling approaches. *J. Anim. Ecol.*, 82, 39-54.
